# Evaluation of Endothelial Progenitor Cell Characteristics as Clinical Biomarkers for Elderly Patients with Ischaemic Stroke

**DOI:** 10.1007/s12015-023-10544-y

**Published:** 2023-05-02

**Authors:** Kamini Rakkar, Othman Ahmad Othman, Nikola Sprigg, Philip M. Bath, Ulvi Bayraktutan

**Affiliations:** Academic Unit of Mental Health and Clinical Neuroscience, Clinical Sciences Building, School of Medicine, The University of Nottingham, Hucknall Road, Nottingham, NG5 1PB UK

**Keywords:** Endothelial progenitor cells, Biomarker, Ischaemic stroke, Angiogenesis, Vasculogenesis

## Abstract

Ageing impairs endothelial function and predisposes the person to ischaemic stroke (IS). Endothelial progenitor cells (EPCs) repair endothelial damage and induce post-ischaemic neovascularisation. Given the prevalence of IS in older population, this study explored whether changes in EPC number and function may reliably predict the type or outcome of stroke in patients ≥ 65 years of age. For this, blood samples were collected once from healthy volunteers (HVs, *n* = 40) and four times (admission and days 7, 30 and 90 post-stroke) from participants with lacunar (*n* = 38) or cortical (*n* = 43) stroke. EPCs were counted with flow cytometry and defined as non-haematopoietic cells (CD45-) expressing markers for stemness (CD34 +), immaturity (CD133 +) and endothelial maturity (KDR +). Clonogenesis, tubulogenesis, migration and proliferation assays were performed as measures of EPC functionality. Biochemical profile of plasma inflammatory and angiogenic agents were studied using specific ELISAs. Primary outcome was disability or dependence on day 90 post-stroke, assessed by the modified Rankin Scale (mRS). Compared to HVs, EPC numbers were higher in stroke patients at all time points studied, reaching significance at baseline and day 30. No differences in EPC counts and functionality were observed between lacunar and cortical stroke groups at any time. Plasma endostatin, PDGF-BB, TNF-α and VEGF levels were higher in stroke patients vs HVs. Patient outcome, evaluated by mRS on day 90 post-stroke, did not correlate with EPC count or functionality. Baseline EPC counts may serve as a diagnostic marker for stroke but fail to distinguish between different stroke subtypes and predict post-stroke outcome.

**Graphical Abstract:**

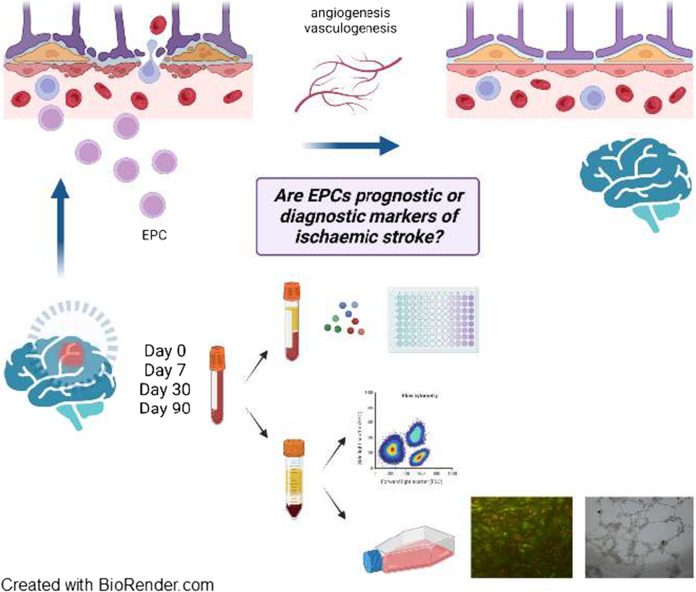

## Introduction

Ischaemic stroke (IS), a leading cause of mortality and morbidity in the world, develops through an interference in blood supply to the brain. The incidence of stroke doubles every 10 years after the age of 55 in both men and women and about 75% of all stroke patients are older than 65 years [1]. Although various cellular and molecular mechanisms may promote atherosclerotic disease during ageing process, endothelial dysfunction, associated with thrombosis and inflammation, is regarded as the main pathology behind much of the structural and functional changes in vasculature [2]. Endothelial dysfunction constitutes the main cause of vascular abnormality in most lacunar strokes that result from an occlusion of a penetrating artery deep within the brain [3-5]. Cortical strokes, on the other hand, develop from an embolism from the heart or large arteries and present a different disease phenotype [6, 7]. The endothelium, composed of endothelial cells (ECs), is tasked with the maintenance of vascular homeostasis, therefore it is important to preserve its integrity and function at all times to prevent age- and stroke-related vascular damage [8, 9].

Bone marrow-derived endothelial progenitor cells (EPCs) play a critical role in sustaining appropriate endothelial function by re-endothelialisation of blood vessels in adult brain and ischaemic settings [2, 10]. Similar to embryonic angioblasts, EPCs possess an inherent capacity to circulate, proliferate and differentiate. Hence, non-haematopoietic cells co-expressing markers for immaturity, stemness and endothelial maturity are regarded as the true EPCs in circulation [11-13]. EPCs can also negate the deleterious effects of cerebral ischaemia by inducing angiogenesis and vasculogenesis which are also adversely affected by the ageing process [12-15].

In light of the above and considering the aetiological differences between lacunar and cortical strokes, we hypothesised that variations in EPC number and function might correlate with patients’ clinical outcome and therefore serve as diagnostic and/or prognostic markers for stroke or its particular subtypes. In addition, the study also investigated the plasmatic profile of inflammatory cytokines, growth factors and/or angiogenic factors known to affect EPC characteristics. One of the greatest obstacles, cause of variation and often ambiguous results in EPC research is the definition and characterisation of EPCs. Therefore, in this study we have sought a comprehensive and exclusive characterisation of EPCs where only non-haematopoietic cells are included in the classification as well as looking at various functional aspects. To our knowledge, this is the first study to scrutinise the comprehensive numerical and functional aspects of EPCs as well as the patient biochemical plasma profile, in two causally distinct subtypes of IS only in participants over the age of 65 years, which represent the age group at greatest risk.

## Materials and Methods

### Participants

This was a single centre, observational, case-controlled study, performed in accordance with the ethical standards for human experimentation established by the Declaration of Helsinki. The study protocol was approved by the West Midlands—Coventry & Warwickshire Research Ethics Committee (REC number: 16/WM/0304) [16]. The study enrolled 81 older patients (≥ 65 years) with lacunar or cortical stroke between February 2017 and November 2019. IS was defined as a sudden focal neurological deficit persisting longer than 24 h with no evidence of cerebral haemorrhage on imaging. Demographic and medical data were collected at baseline. Based on the location of the ischaemic lesions, the patients were divided into two groups. Cortical group included patients with infarcts located in frontal, parietal, or temporal lobes. Lacunar group included patients with small subcortical infarcts and without clinical evidence of cortical dysfunction, e.g. aphasia and hemianopia and whose CT/MRI did not show a relevant lesion exceeding 1.5 cm in size. Forty older HVs (≥ 65 years) with no history of stroke or transient ischaemic attack (TIA) were also recruited for the study to determine the specific correlation between old age and EPC characteristics. Informed consent was obtained from all participants for their anonymised information to be published in this study. Blood samples were collected only once from HVs and four times from stroke patients to cover different phases of stroke, acute (within the first 48 h of stroke, baseline—BL), subacute (day 7 post-stroke, D7) and chronic (days 30 and 90 post-stroke, D30 and D90, respectively).

National Institutes of Health Stroke Scale (NIHSS) was used to measure neurological deficits at BL, D7, D30 and D90. Functional status of patients were evaluated face to face by the modified Rankin Scale (mRS) and Barthel Index (BI) at D90. As all acute/subacute patients received similar standard care including pharmacotherapy, including antiplatelet, statin and anti-hypertensive drugs and underwent an appropriate rehabilitation programme.

### Blood Sampling and Processing

Thirty millilitres of venous blood samples were collected from participants. Of which, 6 mL were used to count circulating EPCs by flow cytometry where non-haematopoietic cells (CD45-) co-expressing cell surface markers for stemness (CD34 +), immaturity (CD133 +) and endothelial maturity (KDR +) were defined as EPCs [11-13]. The remainder of blood samples were used to isolate and culture mononuclear cells (MNCs). To encourage EPCs to mature into outgrowth ECs (OECs), MNCs were seeded on fibronectin-coated flasks and cultured in specialised media supplemented with endothelial growth factors [17]. Cells that endocytosed DiI-conjugated acetylated low density lipoprotein (DiI-AcLDL) and stained positive for FITC conjugated *Ulex europaeus* agglutinin (FITC-lectin) were considered as OECs and used in subsequent functional assessments [12, 13, 17]. To avoid bias, the researchers who performed laboratory experiments remained blinded to patients and samples throughout the study.

### Endothelial Progenitor Cell Functional Assessments

To better correlate EPC functionality to ageing process and IS subtypes, a series of different functional assessments were performed.

### Clonogenesis Assay

MNCs were seeded on 0.01 mg/ml fibronectin-coated 24-well plates and cultured in endothelial basal medium-2 (EBM-2) containing all the necessary supplements and 20% foetal calf serum. Colonies were counted on day 28 of culture using an eyepiece graticule on a light microscope and translated to number per cm^2^.

### Characterisation of Cultured EPCs

MNCs, incubated in EBM-2 containing 100 ng/ml VEGF protein for 96 h, were co-stained with FITC-lectin (Sigma) and DiI-AcLDL (Molecular Probes).

### Mobility Assay

OECs, incubated in EBM-2 media supplemented with 3.5 mg/ml BSA and 5 μg/ml Calcein-AM (Molecular Probes) for 2 h, were seeded in the upper chamber of a modified Boyden chamber (3 μm BD Falcon HTS FluoroBlok) before assessing their migration to lower chamber containing 50 ng/ml VEGF via a bottom plate reading fluorometer.

### Proliferation Assay

OECs, grown on fibronectin-coated 96-well plates in EBM-2 media, were subjected to serum starvation for 48 h in EBM-2 media containing 0.1% BSA and tetrazolium salts (WST-1). Proliferation was measured at 440 nm by analysis of colour generated through breakdown of WST-1 by mitochondrial dehydrogenase.

### Tubulogenesis

OECs, seeded in 48-well plates pre-coated with growth factor reduced Matrigel (BD Biosci), were cultured for 8 h. Tube formation was defined as the appearance of a circle-like structure and visualised by a light microscope.

### Biochemical Assessments

The biochemical profile of patient and HV plasma samples was scrutinised for major angiogenic promoters and inhibitors and inflammatory cytokines and chemokines using specific ELISAs. Angiostatin, endostatin, granulocyte colony-stimulating factor (GCSF), platelet-derived growth factor-BB (PDGF-BB), stromal cell derived factor-1 (SDF-1), tumour necrosis factor alpha (TNF-α), thrombospondin-1, thrombospondin-2, vascular endothelial growth factor (VEGF) were all measured using ELISA kits according to the manufacturer’s protocols (Bio-techne). Changes in total antioxidant capacity (TAC), determined by the sum of endogenous and food-derived antioxidants, were also measured in plasma using a commercial kit (Abcam) according to the manufacturer’s protocols.

### Statistical Analyses

All analysis were undertaken with SPSS statistics software (version 26, IBM, UK). All results were reported as either means ± SD or median with interquartile range (IQR). Data were tested for normality using the Shapiro–Wilk test. Those with a normal distribution were analysed using the Independent Student’s *t*-test. Data with non-normal distributions were analysed using a Mann–Whitney U test. Categorical data were analysed using the Chi-Square Test of Independence. *P* values of < 0.05 were considered significant. Circulating EPC levels were analysed by Mann Whitney U test between groups. Circulating EPC levels at different time points were compared using the Friedman Test. Linear mixed models were used to analyse the intra-individual courses of parameters over time. Univariate proportional odds logistic regression analyses were used to determine the independent impact of different predictive variables on outcome at D90 post-stroke as measured by the mRS. Missing data was excluded from the analysis.

## Results

### Study Population

**Fig. 1:**
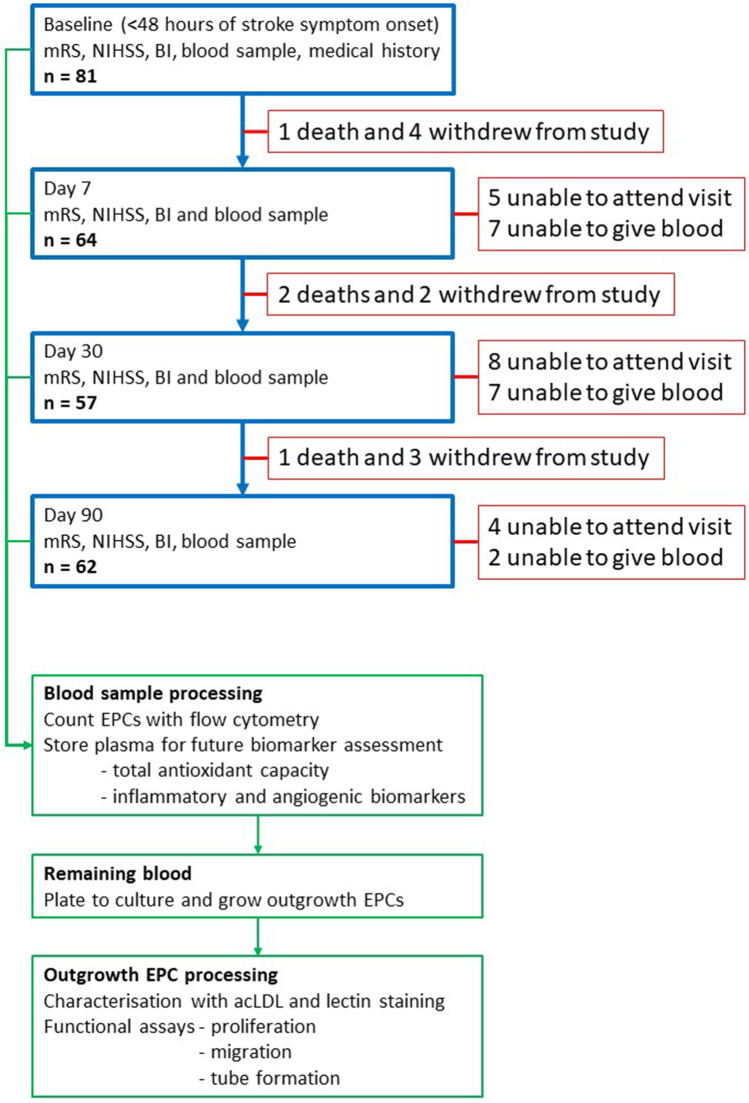
Study recruitment, patient pathway and sample processing. modified Rankin Scale (mRS), National Institution of Health Stroke Scale (NIHSS), Barthel Index (BI), acetylated low-density lipoprotein (acLDL), endothelial progenitor cells (EPCs)

Blood samples were collected from a total of 121 subjects including 40 HVs and 81 patients with lacunar (*n* = 38) or cortical (*n* = 43) stroke. The details of patient recruitment and sample processing are presented in Fig. 1. Throughout the paper, data were analysed to show the potential (dis)similarities between HVs and all IS patients and specifically between cortical and lacunar stroke subgroups.

Despite recruiting only older individuals for the study, the average age, male/female ratio (60% female in HV group and 31% female in stroke group) and the prevalence of cardiovascular risk factors, including hypertension (38% vs 61%) and atrial fibrillation (AF, 5% vs 28%), were significantly higher in IS patients. As expected, compared to HVs, a greater percentage of IS patients were on anti-platelets at baseline. Other than the higher prevalence of hypertension and calcium channel blocker use in cortical group, demographical and medical data collected from patients with cortical and lacunar stroke were similar (Table 1).

**Table 1:** Demographics and clinical features of healthy volunteers (HV) and ischemic stroke patients

	HV (*n* = 40)	Stroke (*n* = 81)	Cortical (*n* = 43)	Lacunar (*n* = 38)	HV vs stroke adjusted *p*-value	cortical vs lacunar *p*-value
Age/years (SD)						
	73.3 (7.2)	76.9 (7.8)	77.3 (7.70)	76.5 (7.9)	**0.015†**	0.644†
Sex (%)						
Female	24 (60.0)	25 (30.9)	16 (37.2)	9 (23.7)	**0.002‡**	0.188‡
Alcohol (IQR)						
Units per week	5.3 (0, 7)	9.4 (0, 12)	11.2 (0, 14)	7.3 (0, 12)	0.369 (0.056*)	0.596*
Smoker (%)						
Current	1 (2.5)	10 (12.5)	6 (14.0)	4 (10.8)	0.148 (0.143‡)	0.913‡
Ex	18 (45.0)	38 (47.5)	20 (46.5)	18 (48.6)		
Never	21 (52.5)	32 (40.0)	17 (39.5)	15 (40.5)		
Stroke questionnaire scores (IQR)						
mRS^§^	-	3 (2, 6)	3 (2, 4)	4 (1.5, 4)	-	0.797*
NIHSS^§^	-	3 (2, 4)	3 (1, 8)	3 (2, 5)	-	0.827*
BI^§^	-	16 (10, 20)	16 (9, 20)	15 (10.5, 20)	-	0.679*
Clinical features						
Systolic BP/mmHg^§^ (SD)	-	158.1 (32.1)	162.8 (32.7)	152.9 (31.0)	-	0.165†
Diastolic BP/mmHg^§^ (SD)	-	79.8 (13.3)	80.4 (14.2)	79.1 (12.3)	-	0.639†
BMI^§^ (SD)	-	26.8 (5.3)	26.9 (5.3)	26.6 (5.4)	-	0.789†
Cholesterol /mmol/L (IQR)	-	4.3 (3.5, 5.1)	4.3 (3.4, 5.2)	4.5 (3.6, 5.1)	-	0.890*
- LDL^§^ (SD)	-	2.42 (0.87)	2.37 (0.94)	2.47 (0.81)	-	0.650†
- HDL^§^ (SD)	-	1.41 (0.39)	1.47 (0.40)	1.34 (0.38)	-	0.196†
ESR/mm/hour (SD)	-	8 (2, 16)	8 (2, 12)	8 (2, 17)	-	0.753*
CRP/mg/L^§^ (SD)	-	5 (5, 7)	5 (5, 7)	5 (5, 7)	-	0.890*
Glucose /mmol/L (SD)	-	6.6 (5.6, 8.5)	6.8 (5.8, 8.8)	6.2 (5.4, 8.5)	-	0.317*
WBC count × 10^9^/L (SD)	-	8.4 (6.4 10.0)	8.4 (6.8, 10.1)	7.8 (6.4, 10.0)	-	0.452*
Comorbidities (%)						
Hypertension	15 (37.5)	49 (61.3)	30 (71.4)	19 (50.0)	**0.003 (0.014‡)**	**0.049‡**
Diabetes mellitus	6 (15.0)	17 (21.5)	8 (19.0)	9 (23.7)	0.592 (0.395‡)	0.652‡
Atrial fibrillation	2 (5.1)	23 (28.4)	17 (39.5)	6 (15.8)	**0.020 (0.003‡)**	0.180‡
Hyperlipidaemia	11 (28.9)	24 (29.6)	14 (32.6)	10 (26.3)	0.502 (0.939‡)	0.539‡
Previous TIA^§^	-	15 (18.5)	4 (9.3)	11 (28.9)	-	0.230‡
Previous IS^§^	-	12 (14.8)	8 (18.6)	4 (10.5)	-	0.307‡
PAD^§^	0 (0.0)	2 (2.5)	1 (2.3)	1 (2.6)	0.999 (0.316‡)	0.929‡
CAD^§^	2 (5.0)	4 (4.9)	2 (4.7)	2 (5.3)	0.604 (0.988‡)	0.899‡
DVT^§^	1 (2.5)	2 (2.5)	1 (2.3)	1 (2.6)	0.620 (0.992‡)	0.929‡
Medication (%)						
Statins	15 (37.5)	38 (47.5)	22 (51.2)	16 (43.2)	0.269 (0.298‡)	0.479‡
ACE inhibitor	10 (25.0)	34 (42.5)	22 (51.2)	12 (32.4)	0.164 (0.061‡)	0.910‡
Ca^2+^ channel blocker^§^	4 (10.0)	17 (21.3)	14 (32.6)	3 (8.1)	0.079 (0.126‡)	**0.008‡**
Beta blocker	6 (15.0)	15 (18.8)	9 (20.9)	6 (16.2)	0.361 (0.610‡)	0.590‡
Anticoagulant	2 (5.0)	10 (12.5)	7 (16.3)	3 (8.1)	0.391 (0.197‡)	0.271‡
Antiplatelets	5 (12.5)	33 (41.3)	18 (41.9)	15 (40.5)	**0.009 (0.001‡)**	0.905‡

Data is shown for all ischemic stroke patients and for stratified cortical and lacunar ischemic stroke subgroups. Data is represented as mean (± SD), median (IQR) or number (%). *P* values for the HV vs stroke comparisons adjusted for age and sex (using binary logistic regression) are shown with unadjusted values in brackets

Values shown in bold represent statistically significant differences

*mRS* § modified Rankin Scale; *NIHSS* National Institutes of Health Stroke Scale; *BI* Barthel index; *BP* Blood pressure; *BMI* Body mass index; *LDL* Low density lipoprotein; *HDL* High density lipoprotein; *ESR* Erythrocyte sedimentation rate; *CRP* C-reactive protein; *WBC* White blood cell; *TIA* Transient ischaemic attack; *IS* Ischaemic stroke; *PAD* Intracerebral, peripheral artery disease; *CAD* Coronary artery disease; *DVT* Deep vein thrombosis; and *Ca*^*2*+^ Calcium

*Mann–Whitney U test, † Student’s t-test, ‡ Chi-square test

### Analyses of EPCs as Predictors of Stroke Subtype

**Fig. 2:**
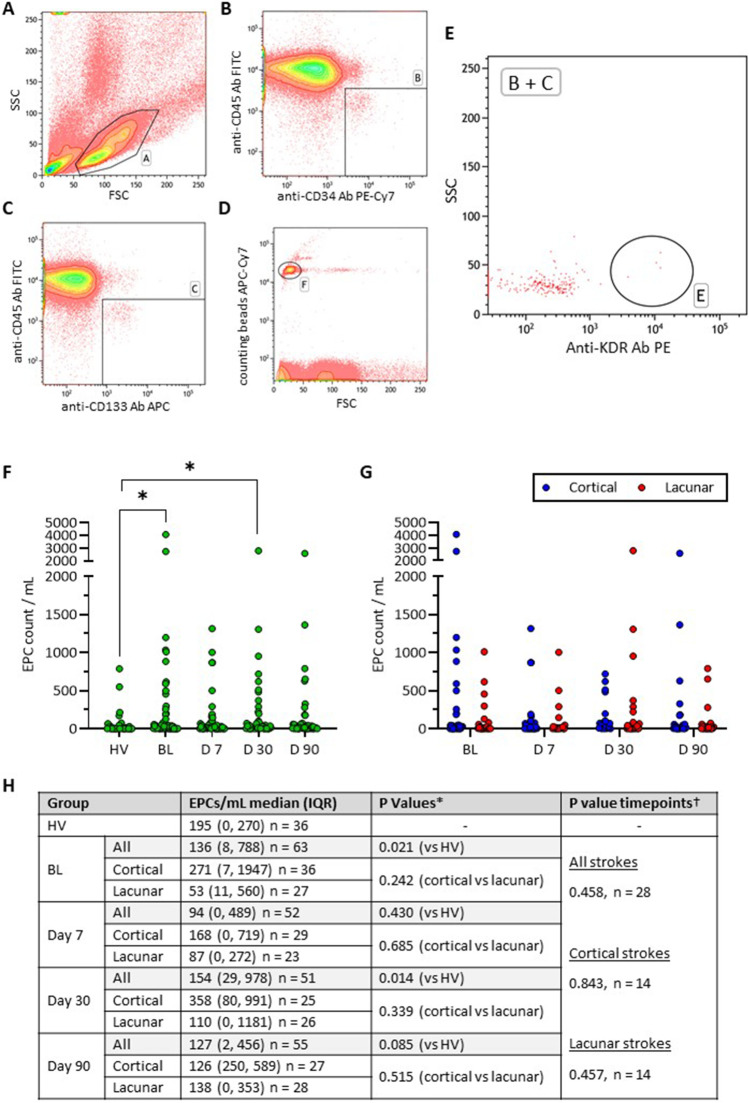
Endothelial progenitor cell (EPC) counts. **(A-E)** EPCs were detected by flow cytometry and analysed using Kaluza Analysis 2.1 software. Compensation controls were used in setting up the flow protocol. Fluorescence minus one and isotype controls were used in gating for positive cells. CD45-FITC, CD34-PECy7, CD133-APC and KDR-PE. Approximately 1 million cells were counted. Cells were first gated on the monocyte and lymphocyte gate [A], then for CD45^−^ CD34^+^ [B] and CD45^−^ CD133^+^ [C] cells. Finally, cells were gated for [B] + [C] and KDR^+^ cells [E]. Counting beads were used to normalise samples to EPC counts/mL [F]. **(F)** EPC counts per mL of peripheral blood for health volunteers (HV) and ischemic stroke patients at baseline (BL) and days 7, 30 and 90 post-stroke. **(G)** EPC counts per mL of peripheral blood for ischemic stroke patients stratified into cortical and lacunar sub-groups at BL and days 7, 30 and 90 post-stroke. **(H)** Median EPC counts for the groups in panels F and G above with *p* values from either the Mann–Whitney U test (*) or Friedman Test (†) comparing HVs to all stroke patients at each time point, comparing cortical and lacunar strokes within the same timepoint or comparing all stroke patient timepoints as a group. *P* values of < 0.05 were considered significant and plotted with a *

Numbers of circulatory EPCs, defined as CD45-CD34 + CD133 + KDR + cells, were considerably higher in IS patients at all study time points, but significant only at BL and D30 (Fig. 2A-E). EPC numbers in lacunar and cortical stroke subgroups did not significantly change at any specific time point or when all data combined (Fig. 2F-H).

### Cellular and Functional Characterisation of EPCs

**Fig. 3:**
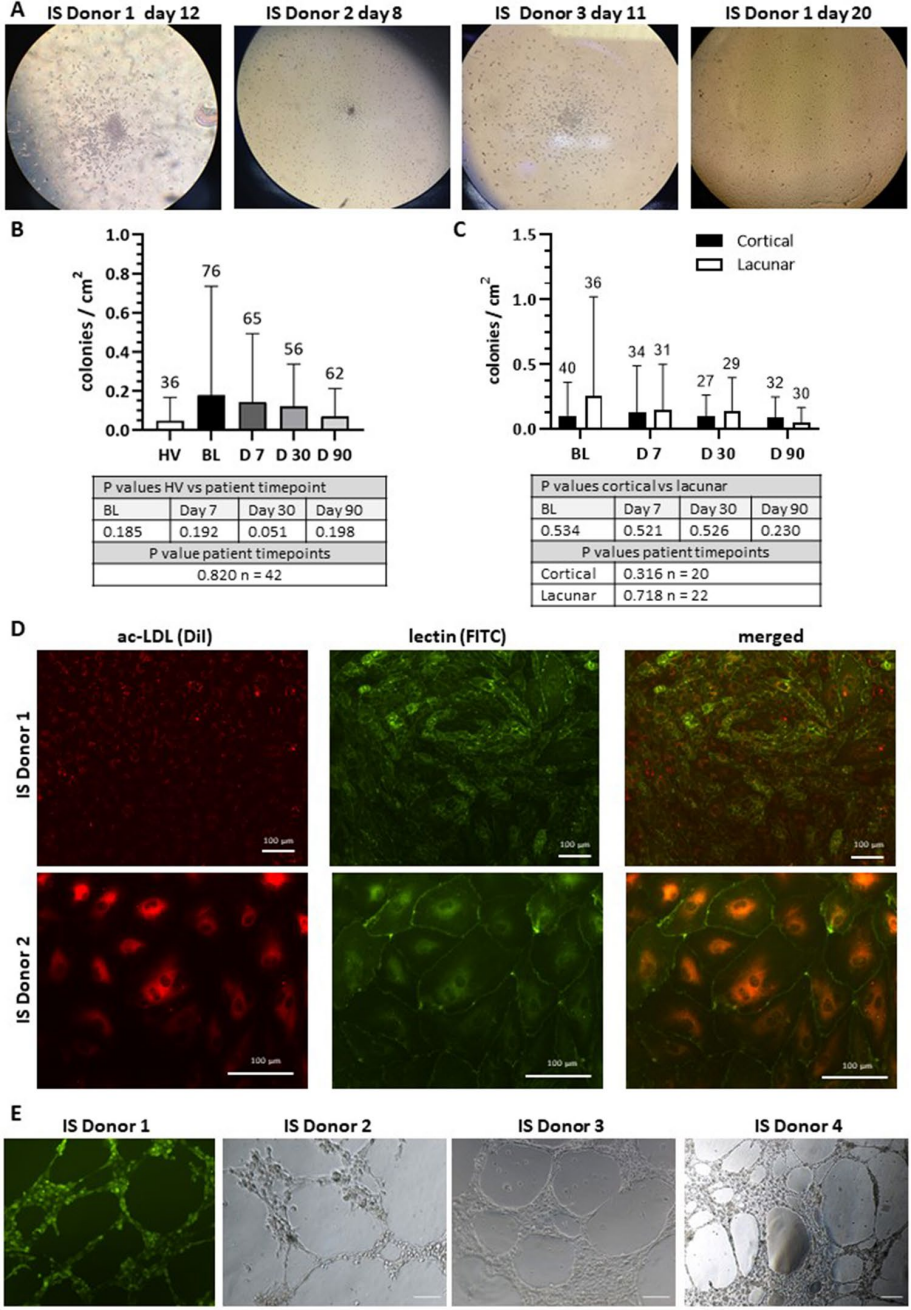
Detection and characterisation of endothelial progenitor cells (EPCs). **(A)** Colony forming units after seeding remaining mononuclear fraction into 24 well plates for 3 different donors. **(B)** No significant differences in the number of EPC colonies per cm^2^ were seen between HV and stroke patients (Mann–Whitney U-test) or between different timepoints post-stroke (Friedman Test). **(C)** No significant differences in the number of EPC colonies per cm^2^ were seen between cortical and lacunar stroke patients at any timepoints post-stroke (Mann–Whitney U-test) or between timepoints in the subgroups themselves (Friedman Test). **(D)** Staining outgrowth endothelial cells with acetylated low-density lipoprotein (ac-LDL) and lectin for 2 different donors. Positive staining indicates endothelial cells. **(E)** Matrigel tube formation assay from 4 different donors shown with either calcein-AM staining or under phase contrast

Examination of the propensity of EPCs to form colonies and proliferate revealed similar colony numbers for HVs and IS patients and for lacunar and cortical stroke patients within four weeks of seeding of MNCs (Fig. 3A-C). Cells sprouted from these colonies formed clearly identifiable tubules on matrigel, endocytosed DiI-AcLDL and stained positive with FITC-lectin, markers for EC maturity (Fig. 3D-E).

**Fig. 4:**
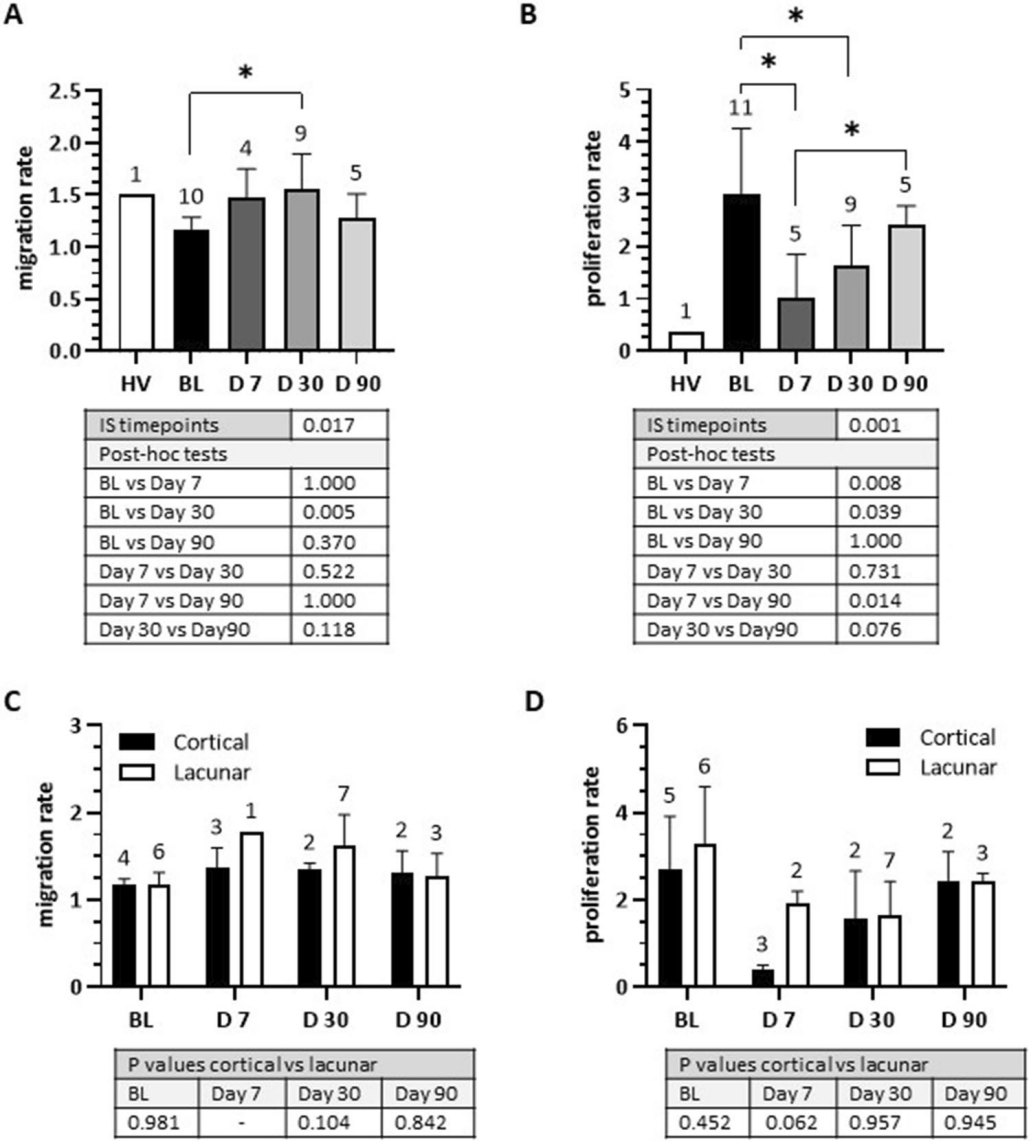
Functional properties of outgrowth EPCs from healthy volunteers (HV) and ischemic stroke patients at baseline (BL) and days 7, 30 and 90 post-stroke. **(A)** The rate of EPC migration was significant between BL and Day 30 post-stroke (Linear mixed model with Bonferroni adjusted *P* value for post-hoc tests). **(B)** The rate of EPC proliferation was significant between BL and Day 7, BL and Day 30 and Day 7 and Day 90 post-stroke (Linear mixed model with Bonferroni adjusted *P* value for post-hoc tests). **(C)** No significant differences in migration rates were seen between cortical and lacunar stroke patients at any timepoints post-stroke (Student’s t-test). **(D)** No significant differences in proliferation rates were seen between cortical and lacunar stroke patients at any timepoints post-stroke (Student’s t-test). *P* values for the statistical tests are shown directly below the plots and n numbers are shown directly above the bars. *P* values of < 0.05 were considered significant and plotted by a *

EPC migration and proliferation rates varied markedly between BL and D30 post-stroke groups. Significant differences were also observed between BL and D7 and D7 and D90 proliferation rates in IS patients (Fig. 4A-B). No differences were observed in EPC migratory and proliferative characteristics between lacunar and cortical stroke patients at any time point (Fig. 4C-D).

### Patients with IS Display Variations in Inflammatory and Angiogenic Modulator Generation

IS patients had consistently higher plasma levels of pro-inflammatory and pro-angiogenic cytokines i.e. TNF-α and VEGF than HVs. Differences in plasma levels of TNF-α were also specifically significant at D30 and D90 post-stroke compared to HVs. Levels of VEGF gradually decreased in stroke patients and on D90 after stroke dropped to the levels seen in HVs. Circulatory levels of TAC and agents known to mediate release, trafficking and homing of EPCs to the site of injury, namely SDF-1 and G-CSF appeared to be unchanged between patients and HVs (Table 2).

**Table 2:**
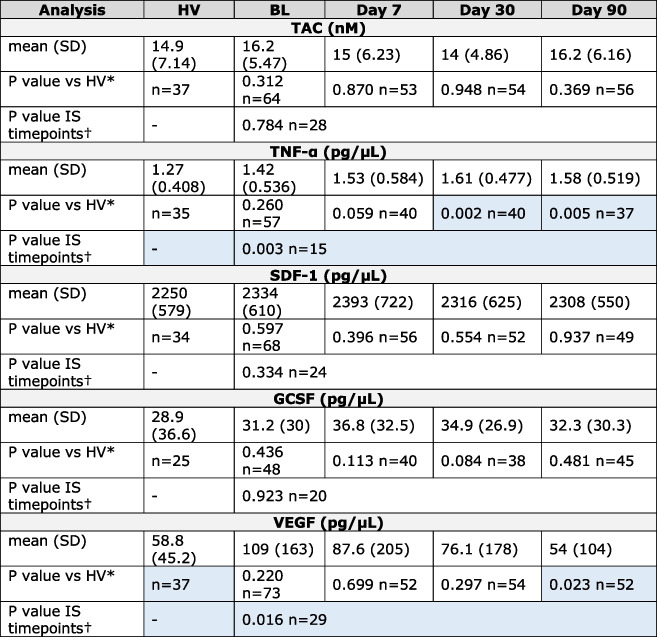

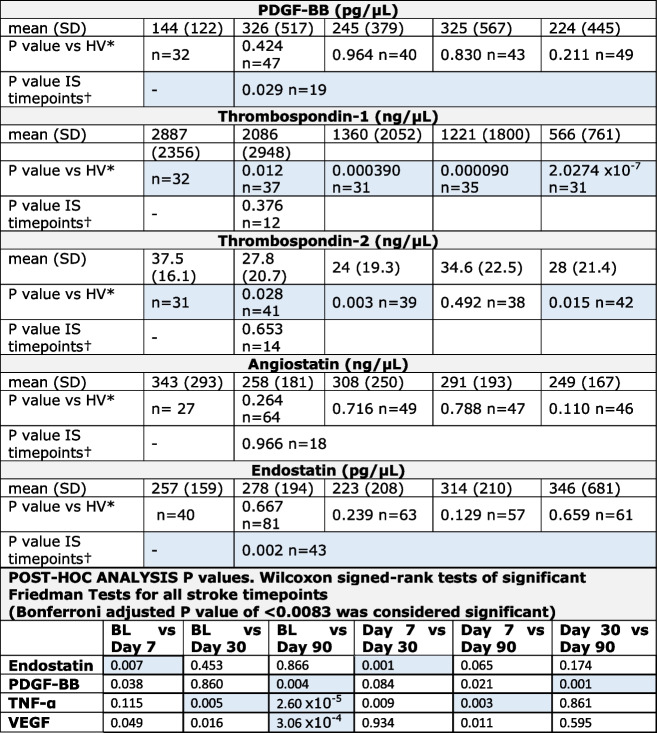
Analysis of biological markers in the plasma of healthy volunteers (HV) and ischaemic stroke (IS) patients at baseline (BL) and days 7, 30 and 90 post ischaemic stroke

Total antioxidant capacity (TAC) was detected in plasma using a kit and values are Trolox equivalent capacity. Angiostatin, endostatin, granulocyte colony-stimulating factor (GCSF), platelet-derived growth factor-BB (PDGF-BB), stromal cell derived factor-1 (SDF-1), tumour necrosis factor-α (TNF-α), thrombospondin-1, thrombospondin-2 and vascular endothelial growth factor (VEGF) levels in plasma were detected using ELISAs. A *P* value of < 0.05 was considered significant (highlighted in blue)

^*^Mann–Whitney U test, †Friedman Test

Contrary to proangiogenic cytokines, the plasma levels of anti-angiogenic factors, thrombospondin-1 and thrombospondin-2 were significantly lower at each study time point (apart from D30 for thrombospondin-2) in IS patients compared to HVs. In contrast, no difference was observed in angiostatin or endostatin levels in IS patients compared to HVs (Table 2).

**Fig. 5:**
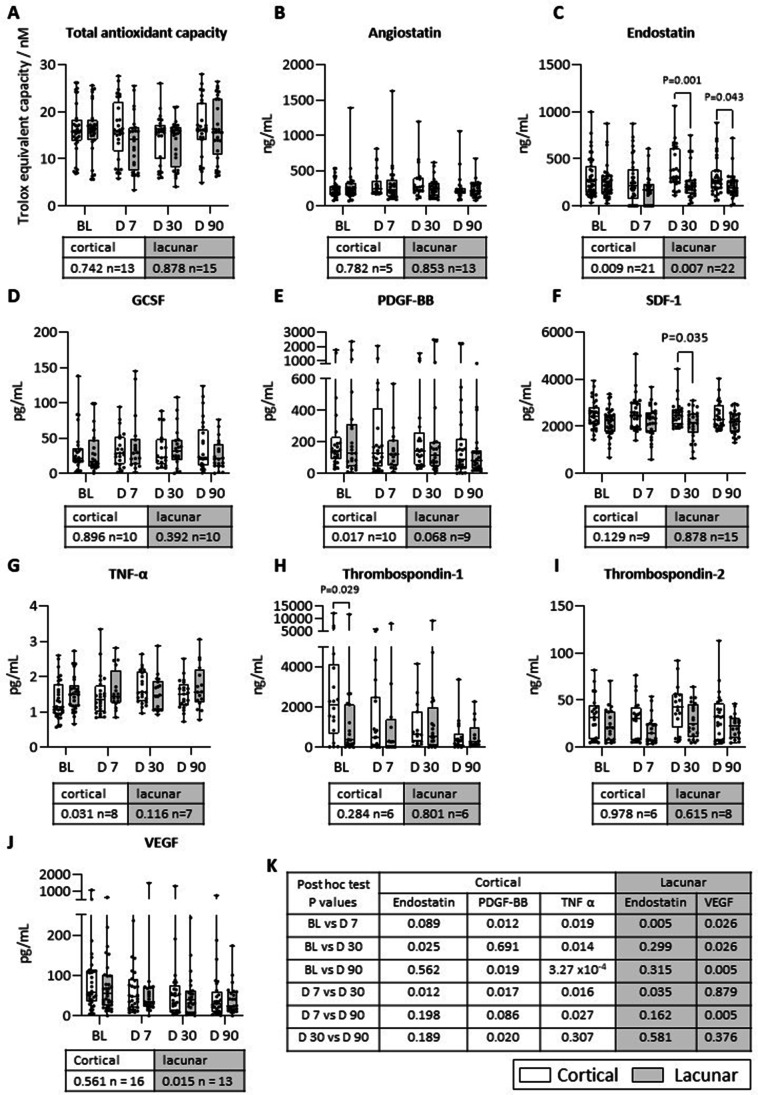
Analysis of biological markers in the plasma of ischaemic stroke patients stratified to cortical and lacunar subgroups at baseline (BL) and days 7, 30 and 90 post ischaemic stroke. **(A)** Total antioxidant capacity (TAC) was detected in plasma using a kit and values are Trolox equivalent capacity. **(B)** Angiostatin, **(C)** endostatin, **(D)** granulocyte colony-stimulating factor (GCSF), **(E)** platelet-derived growth factor-BB (PDGF-BB), **(F)** stromal cell derived factor-1 (SDF-1), **(G)** tumour necrosis factor alpha (TNF-α), **(H)** thrombospondin-1, **(I)** thrombospondin-2 and **(J)** vascular endothelial growth factor (VEGF) levels in plasma were detected using ELISAs. A Mann–Whitney U test was used to compare data from cortical and lacunar sub-groups at the same timepoint and a *P* value of < 0.05 was considered significant and plotted above. A Friedman Test was used to compare all timepoints of the same subgroups (*P* values directly under plot) with a post-hoc Wilcoxon signed-rank test where a Bonferroni adjusted *P* value of < 0.0083 was considered significant. **(K)** Post-hoc analysis *P*-values

Analyses of the abovementioned parameters in patient subgroups at different study time points showed significant increases in endostatin (D30 and D90), thrombospondin-1 (BL) and SDF-1 (D30) levels in cortical vs lacunar subgroup. Comparison of all time points of the same subgroups showed significant changes in endostatin, PDGF-BB and TNF-α levels in cortical and in endostatin and VEGF levels in lacunar patient subgroups (Fig. 5A-J). Post-hoc analysis of these variations show that in the cortical subgroup there was a significant increase in TNF-α levels from BL to D90 and in lacunar subgroup there was a significant decrease in both endostatin levels from BL to D7 and VEGF levels from BL to D90 and D7 to D90.

### Attempts to Identify Prognostic Marker(s) for IS

**Fig. 6:**
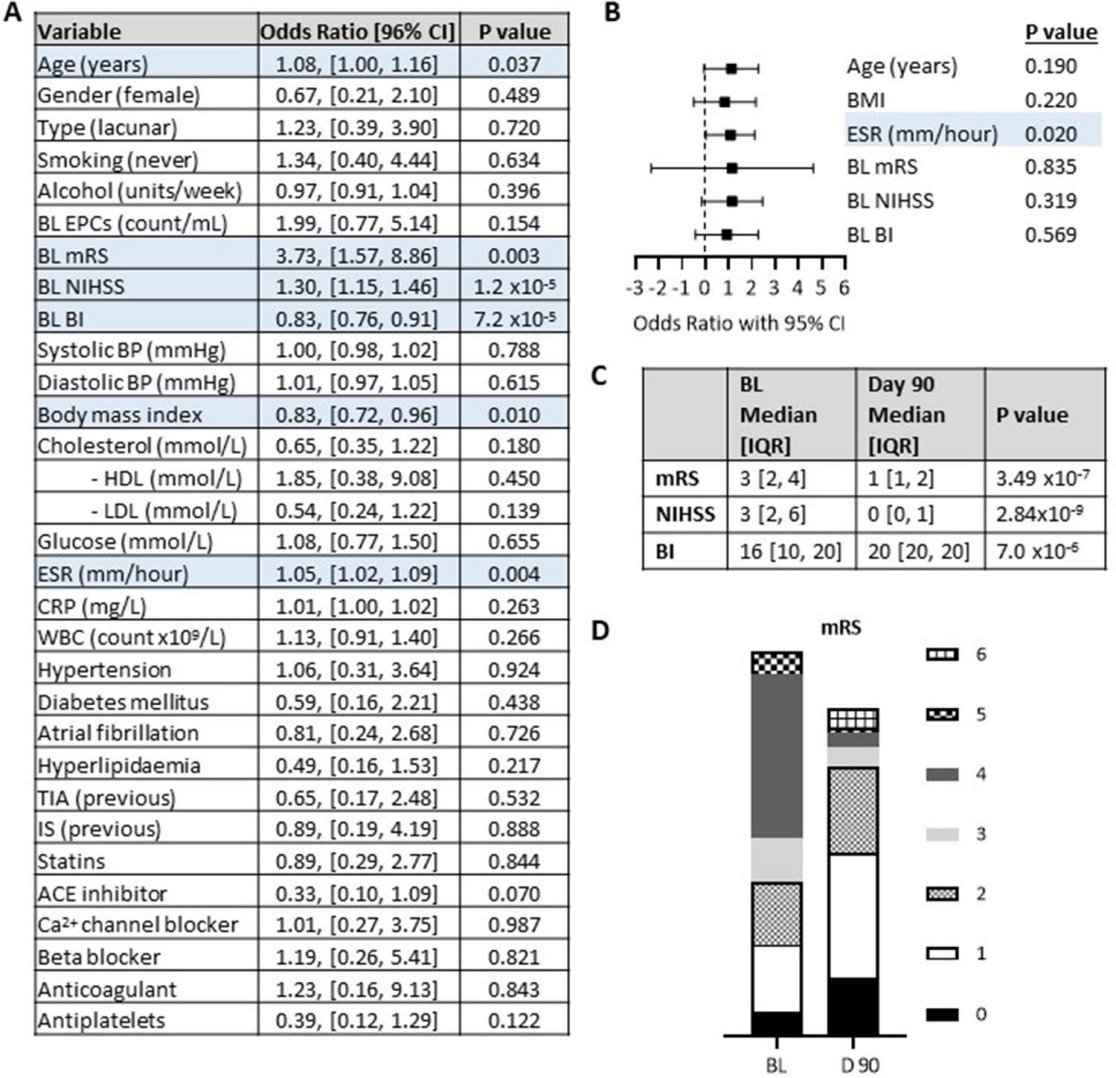
Predictors of day 90 outcome as measured by the modified Rankin Scale (mRS). **(A)** Univariate proportional odds logistic regression of baseline (BL) variables to predict outcome at day 90 post ischaemic stroke as measured by the mRS. The day 90 mRS data was categorised into the following bands 0–2, 3–4 and 5–6 to represent mild, moderate and severe disease. **(B)** All variables which were significant on univariate analysis were entered into the model. **(C)** Wilcoxon signed-rank tests were used to compared baseline and day 90 data for mRS, NIHSS and BI outcome measures and *P* values are displayed in the table. **(D)** Frequency distribution of patients in mRS categories at baseline and day 90. *P* < 0.05 was considered significant for all tests. Endothelial progenitor cells (EPC), National Institution of Health Stroke Scale (NIHSS), Barthel Index (BI), blood pressure (BP), low density lipoprotein (LDL), high density lipoprotein (HDL), erythrocyte sedimentation rate (ESR), C-reactive protein (CRP), white blood cell (WBC), transient ischemic attack (TIA), ischemic stroke (IS) and calcium (Ca^2+^)

Univariate proportional odds logistic regression of multiple baseline variables was conducted on the cortical and lacunar IS subgroup together as one cohort due to lack of statistical power in separate subgroups. It identified age, body mass index (BMI), erythrocyte sedimentation rate (ESR) and baseline mRS, NIHSS and BI as predictors of stroke outcome at D90 as measured by the mRS (Fig. 6A). These significant univariate predictors were evaluated in a multivariate proportional odds logistic regression model and results suggested only ESR as prominent predictor (Fig. 6B) in the multivariate model.

Comparison of patients’ neurological outcomes at BL and D90 has revealed that most patients recovered well and regained independence as confirmed by mRS, NIHSS and BI assessments. Indeed, frequency distribution of patients in mRS categories at BL and D90 reveal that while most patients had mRS score of ≥ 3 at BL, indicator of a moderate disease, at D90 most patients had a score of ≤ 2, indicating independence (Fig. 6C-D).

## Discussion

EPCs replace dead/dying ECs to restore vascular equilibrium and this study investigated whether EPC number and/or function post-stroke can be used as diagnostic or prognostic markers for IS and if there are any differences by IS subtype (lacunar or cortical). Despite aetiological differences, the former is associated with endothelial dysfunction atherosclerosis and small arterial occlusions and infarcts deep within the white matter while the latter is associated with large vessel occlusion and grey matter infarcts [18, 19], post-stroke imaging remains the mainstay for the diagnosis of stroke subtypes. Furthermore ageing represents the strongest risk factor for IS and is associated with greater mortality and poorer quality of life in people ≥ 65 years of age [1, 16]. Therefore, this study examined the diagnostic or prognostic value of these cells in participants over the age of 65 years. Overall this study has found EPC cannot be used as diagnostic or prognostic marker for IS and there are no differences in EPC number or function between lacunar and cortical strokes.

In line with recent publications, non-haematopoietic cells (CD45-) co-expressing CD133, CD34 and KDR antigens were defined as EPCs whose release appeared to be induced by ischaemic injury at all study time points, reaching significance only at BL and D30 [11-13]. These indicate that EPCs may, in some sense, serve as diagnostic marker for acute IS. Sharp (within 24 h) or gradual (peaking on D7) increases as well as decreases in EPC counts have been reported in previous studies [20-22]. However, employment of different antigens, timepoints or methods (colony forming units/flow cytometry) to detect/quantify EPCs in different studies somewhat explains these discrepancies [22, 23]. EPCs represent a rare cell population in the circulation and are often undetected in participants [24]. This results in data bias and difficulty in analysing results and ultimately different statistical analysis methods are employed across studies. Our current study classifies EPCs with the most comprehensive set of antigen markers and functional assessments similar to that used in a recent study where EPC numbers were also increased after IS albeit at different timepoints (3 and 12 months).

Observation of similar EPC counts in patients with lacunar or cortical stroke throughout the study rules out the use of “EPC counts” as reliable diagnostic biomarkers for these IS subtypes. Although EPC numbers were previously reported to be lower in patients with large- versus small-vessel disease on day 1 post-stroke, this study was quite limited in its scope i.e. covered only the acute phase of the disease [25] and also classified EPCs based on only 2 antigen markers instead of 4 as used in this study. Taken together, the data presented so far propose ischaemic injury, rather than the location and size of the damaged neurovasculature, as the main driver of EPC release.

Observation of similar colony numbers on HVs and IS subgroup plates indicated the similar tendency of all progenitor cells for proliferation and lineage differentiation. Inherently higher proliferative and migratory tendencies of progenitor cells may be crucial to elevate the number of EPCs at the site of vascular damage and thereby restore neurovascular integrity and function. Increased angiogenesis, reduced infarct/oedema volumes and improved long term neurological outcome observed in MCAO rats transplanted with EPCs corroborate this notion [26]. Albeit requiring further confirmation, OECs from IS patients showed increased proliferative but slightly reduced migratory capacity compared to their counterparts from HVs. It is possible that while sporadic injuries affecting few vascular ECs may benefit from a directed speedy migration of few EPCs in HVs, relatively larger or widespread injuries in stroke patients may require substantially higher numbers of EPCs to effectively repair cerebrovascular damage thereby obviating the need for directed rapid migration. Observation of similar OEC migratory and proliferative rates in lacunar and cortical stroke subgroups at all study time points imply that ischaemia-mediated focal vascular damage, rather than the size of the affected artery, influences these characteristics.

Biochemical analyses of HV and IS patient plasma samples revealed consistent increases in pro-inflammatory cytokine, TNF-α in IS patients. This confirmed a shift to pro-inflammatory state after stroke [27, 28]. However, increased availability of pro-angiogenic cytokine, VEGF and the diminished availability of anti-angiogenic factors that normally inhibit the activity of VEGF i.e. thrombospondin-1/2 may counter the effects of TNF-α and induce angiogenesis especially during the early phases of IS to minimise the extent of neurovascular damage [29]. Similarities in circulating levels of SDF-1 and G-CSF between HVs and IS patients, known to mediate the release and homing of EPCs to the site of injury, would suggest that VEGF, which is increased in IS patients compared to HVs, is the main angiogenic factor post-stroke [30]. Analyses of these parameters in specific patient subgroups showed time-dependent increases in thrombospondin-1 (BL), endostatin (D30 and D90) and SDF-1 (D30) levels in cortical versus lacunar subgroup. Confirming that although angiogenesis is constantly regulated, the degree of variations and the time points on which they appear dismiss any of the abovementioned angiogenic or pro/anti-inflammatory cytokines as reliable diagnostic markers.

It is likely that environmental changes evoked by ischaemic injury and ageing per se may also suppress the generation and function of EPCs. Oxidative stress, emerging from an imbalance between production and metabolism of ROS, represents one such change and leads to cerebrovascular dysfunction through disruption of major cellular components and neutralisation of nitric oxide, a potent anti-atherogenic agent [9]. Observation of similar plasma TAC, determined by the sum of endogenous and food-derived antioxidants, in HVs and patients and between lacunar and cortical stroke subgroups suggest that there may be imbalances in the availability of ROS in neurovascular impairments [9] but the antioxidant capability between HVs and IS patients remains unchanged. Therefore, in IS patients an increase in or supplement of their endogenous antioxidant capability may be beneficial. For example, in dysfunctional arterial segments, normal vascular function was restored by a series of antioxidants e.g. vitamins and ROS scavengers, which also affirms the role of oxidative stress in endothelial dysfunction [31].

As alluded above, increased post-ischaemic release of EPCs may indicate a better patient outcome [25] however in this study EPC number and function at baseline was not a predictor of patient outcome. As the majority of patients enrolled for the study had significant recovery on D90, evidenced by mRS scores which were mostly above 3 at BL and below 3 at D90, it is essential to investigate these links in patients who continue to show dependence on D90.

These results would benefit from replication in another larger cohort. In this study patient recruitment and retention was a limitation. The prospective aim was to recruit 100 patients; however this target was not reached with only 81 patients recruited. Furthermore, patient retention at post-stroke visits was also low with only 62 visits completed at day 90. Collectively, this has resulted in lower statistical power and could also have biased the results. Also, although not directly investigated in this study, wider use of antithrombotics and anti-hypertensives in IS versus HV group, due to higher prevalence of AF and hypertension, may somewhat explain the abundance of circulating EPC after IS [32, 33] and these results would need further investigation.

In conclusion, levels or function of EPCs did not correlate with patients’ outcome in this study and therefore they did not serve as prognostic markers. The number of EPCs did increase after IS and therefore could be used as a diagnostic marker but would offer little benefit over current diagnostic methods. However increased EPC counts, capable of inducing angiogenesis and vasculogenesis, may serve as diagnostic marker for other events involving vascular injury or dysfunction. Further research into EPCs as biomarkers to distinguish between IS and stroke mimics or TIAs would be beneficial.

## Data Availability

The datasets generated during and/or analysed during the current study are available from the corresponding author on reasonable request.

## Declarations

### Clinical Trial Registration

NCT02980354
